# *Hadhb* Deficiency Inhibits Lung Tumorigenesis Via Activating ER Stress

**DOI:** 10.7150/jca.130384

**Published:** 2026-05-01

**Authors:** Wuhou Dai, Dong Xu, Yubin Lei, Shujing Zhang, Tonglu Yu, Wufan Tao, Xiaofeng Chen, Xingrong Du

**Affiliations:** 1 Huashan Hospital and School of Life Sciences, Fudan University, Shanghai, P.R. China.; 2 Huashan Hospital, Fudan University, Shanghai, P.R. China.; 3 Shanghai Key Laboratory of Metabolic Remodeling and Health, Institute of Metabolism and Integrative Biology, Drug Clinical Trial Center, Shanghai Xuhui Central Hospital/Xuhui Hospital, Fudan University; Institute of Clinical Mass Spectrometry, Shanghai Academy of Experimental Medicine, Shanghai, P.R. China.

**Keywords:** *Hadhb*, lung cancer, ER stress, *Chop*

## Abstract

Lung cancer remains the leading cause of cancer-related mortality worldwide, yet its molecular pathogenesis is not fully understood. Here, we identify *HADHB,* encoding a mitochondrial β-ketothiolase as a novel oncogene in lung cancer. Using a *Hadhb^PB/PB^* mouse model, we demonstrate that *Hadhb* deficiency in mice significantly suppresses *K-Ras*^G12D^-driven lung tumor progression by impairing cancer cell proliferation and enhancing apoptosis. Mechanistically, *Hadhb* loss induces proteotoxic endoplasmic reticulum (ER) stress, as evidenced by increased ATF6(N) and phosphorylation of eIF2α and subsequent upregulation of the pro-apoptotic factor CHOP. Crucially, knockdown of *Chop* partially restores oncogenic potential in *Hadhb^PB/PB^* mice and rescues the growth defect of *HADHB*-depleted cells, establishing CHOP as a key downstream mediator. Consistent with its oncogenic role, HADHB protein is upregulated in 52% (19/36) of human lung tumors compared to adjacent normal tissues, and low *HADHB* mRNA levels are correlated with good prognosis in lung cancer patients. Our findings unveil a previously unrecognized *HADHB*-*CHOP* regulatory axis in lung cancer progression and provide preclinical rationale for targeting mitochondrial metabolism in combination with ER stress modulators as a therapeutic strategy.

## Introduction

The mitochondrial trifunctional protein (MTP) is a hetero-octameric complex located on the inner mitochondrial membrane, comprising four α-subunits (HADHA) and four β-subunits (HADHB). It catalyzes the final three steps of long-chain fatty acid β-oxidation (FAO) 1. The α-subunit harbors both long-chain enoyl-CoA hydratase and 3-hydroxyacyl-CoA dehydrogenase activities, while the β-subunit provides the long-chain 3-ketoacyl-CoA thiolase activity essential for the terminal cleavage reaction 1. Mutations in either *HADHA* or *HADHB* cause MTP deficiency, a rare autosomal recessive disorder with a broad clinical spectrum ranging from severe, often lethal, neonatal cardiomyopathy to a milder, late-onset sensorimotor polyneuropathy 2-4. Reflecting this clinical heterogeneity, *Hadha* knockout mice died neonatally from hypoglycemia 5, whereas *Hadhb* point-mutant mice developed late-onset cardiac fibrosis and hepatic steatosis, succumbing to sudden death between 9-16 months of age 6.

Beyond its role in inherited metabolic disease, fatty acid β-oxidation is increasingly recognized as a critical energy and biosynthetic source for rapidly proliferating cancer cells, supporting membrane synthesis, signaling, and redox homeostasis 7-9. The role of MTP in cancer, however, appears context-dependent. In clear cell renal cell carcinoma and colorectal cancer, low or altered *HADHB* expression is associated with poor prognosis and chemo-resistance, respectively 10-12. Conversely, high *HADHB* expression correlates with poor survival in head and neck squamous cell carcinoma and high-grade lymphomas 13, 14, highlighting its potential tissue-specific functions. Despite these associations, the functional significance of *HADHB* in lung cancer pathogenesis remains poorly characterized.

Endoplasmic reticulum (ER) stress represents a key node linking cellular metabolism and survival. The accumulation of misfolded proteins or toxic lipids disrupts ER homeostasis, triggering the unfolded protein response (UPR) 15-17. This adaptive signaling is mediated by three ER-resident sensors—PERK, IRE1, and ATF6—which collectively work to restore proteostasis. However, persistent ER stress commits cells to apoptosis, predominantly through the induction of the pro-death transcription factor CHOP 18. The role of ER stress in cancer is controversial. Several observations indicate that the UPR signaling can promote angiogenesis by regulating the transcription of several pro-angiogenic factors 19, 20. On the other hand, UPR-mediated apoptosis has been proposed as a strategy for ameliorating cancer 21-23. In spite of that, its role in lung tumorigenesis remains elusive.

Here, we identify *HADHB* as a novel oncogene in lung cancer. We found that HADHB is upregulated in human lung tumors and that its elevated mRNA level predicts poor patient prognosis. Genetic ablation of *Hadhb* potently attenuated tumor development in a *K-Ras*-driven mouse model, concomitant with suppressed proliferation and increased apoptosis. Mechanistically, *Hadhb* deficiency induced chronic ER stress, as evidenced by robust upregulation of CHOP in both murine tumors and HADHB-depleted human lung cancer cells. Crucially, knockdown of *CHOP* partially rescued the tumor-suppressive effects of *Hadhb* mutation *in vivo*. Our findings unveil a metabolic-stress regulatory axis in which H*ADHB* promotes tumor survival by restraining ER stress-induced, CHOP-mediated apoptosis, revealing new therapeutic avenues for lung cancer.

## Materials and Methods

### Animals

*Hadhb* PB (*PiggyBac*) mutant mice were generated on the FVB/NJ background in Institute of Developmental Biology and Molecular Medicine, Fudan University 24. LSL*-K-Ras^G12D^* mice (thereafter called *K-Ras* mice) carrying a LSL*-K-Ras^G12D^* allele in FVB genetic background were previously described 25. *Hadhb^PB/PB^* mice were crossed with *K-Ras* mice to generate *Hadhb^+/PB^ K-Ras* mice and *Hadhb^PB/PB^ K-Ras* mice. All mice were maintained on 12/12-h light/dark cycles. Experiments were conducted with consent from the Animal Care and Use Committee of the Institute of Developmental Biology and Molecular Medicine at Fudan University, Shanghai, China.

### Lung tumor induction, enumeration and tumor burden analysis

Mouse lung tumors were induced as the way described previously 26. Two-months-old control *K-Ras* mice and *Hadhb^PB/PB^ K-Ras* mice were infected with 5×10^7^ plaque-forming units (PFU) of adenovirus expressing CRE recombinase (Ad-*Cre*) by tracheal instillation. Eight to ten weeks after Ad-*Cre* infection, mouse lungs were retrieved and tumors on mouse lung surface were counted under dissection microscope. For tumor burden analysis, lungs were perfused through the trachea with 4% paraformaldehyde and fixed overnight followed by standard procedures for paraffin sections and H&E staining. Six randomly selected lung sections/mice were scanned for at least three mice each genotype. Total lung area occupied by tumor was measured and tumor burden was calculated as previously described by Lei, *et al.* 27. For rescue experiments, control *K-Ras* mice and *Hadhb^PB/PB^ K-Ras* mice were infected with 10^6^ transforming unit (TU) of lentivirus expressing *Cre* and shRNA (lenti-*Cre-*scramble or lenti-*Cre-*shChop) by tracheal instillation. Twelve weeks after lentivirus infection, mouse lung tumor development was evaluated the same as described above for adenovirus-infected mice.

### TUNEL assay and EdU incorporation assay

TUNEL assay was performed following the manufacturer's instructions (#11684795910, Roche). For EdU (5-ethynyl-2′-deoxyuridine) assay, mice were injected intraperitoneally with 50 mg/kg EdU eight weeks after virus infection. Two days after EdU injection, lung tissues were retrieved and subjected to frozen section followed by EdU staining with a kit (#C10310, RiboBio, Guangzhou, China) according to manufacturer's instruction. At least five lung sections/mice were used for EdU staining for three mice each genotype. EdU positive cells on four randomly selected fields/section were calculated for statistical analysis.

### Plasmids

For pLKO.2 plasmid, the BamH I-Kpn I puromycin fragment in pLKO.1 was replaced by an EGFP fragment. For pLKO.1-scramble and pLKO.1-shHADHB, the scramble and shHADHB oligos were separately cloned into the Age I and EcoR I sites of pLKO.1. For pLKO.2-scramble and pLKO.2-shCHOP plasmid, the scramble and shCHOP oligos were separately cloned into the Age I and EcoR I sites of pLKO.2. For plasmid pFUW-*Cre*-scramble and pFUW-*Cre*-shChop: U6 promoter and shRNA oligos were cloned at the end of *Cre* gene in pFUW-*Cre* 27. For plasmid pCD513B-dnAMPK, the cDNA for a dominant negative catalytic subunit (α1) of AMPK (dnAMPK) was generated as described 28 and then cloned into Nhe I-Not I sites of pCD513B. The shRNA oligos were listed below:

### Virus production and cell culture

Mouse Lewis Lung Carcinoma cell line was maintained in high-glucose DMEM supplemented with 10% fetal bovine serum (FBS). Human cancer cell lines HT-29, H460, H522, H23, H1650 and A549 were cultured in RPMI-1640 with 10% FBS. All the cell lines were purchased from the National Collection of Authenticated Cell Cultures (Shanghai, China). The preparation of Ad-*Cre* was described by Lei, *et al.* 27. Recombinant lentiviruses (lenti-shHADHB, lenti-dnAMPK, and lenti-shCHOP) were produced by co-transfecting HEK293T cells with the respective expression plasmids (pLKO.1-shHADHB, pCD513B-dnAMPK, or pLKO.2-shCHOP) and the packaging plasmids pCMV-VSV-G, pRSV-Rev, and pMDLg/pRRE. Culture supernatants containing lentiviruses were harvested 48 hours post-transfection and either used directly for infection or stored at -80°C.

For stable gene knockdown, H460, H522 and HT-29 cells were infected with lenti-shHADHB or a lenti-scramble control lentiviruse and selected with 2 µg/mL puromycin for one week. For further rescue experiments, these cells were infected with lenti-shCHOP or lenti-dnAMPK, and EGFP-positive cells were isolated by flow cytometry.

Lenti-*Cre*-scramble and lenti-*Cre*-shChop viruses were generated by co-transfecting HEK293T cells with pFUW-*Cre*-scramble or pFUW-*Cre*-shChop and the same packaging plasmids. Supernatants were harvested after 48 hours and concentrated as previously described 29. The titer (TU) of these viruses was determined by infecting HEK293T cells harboring LSL-EGFP transgenes, following established methods 27. All human cell lines are mycoplasma-free and have been authenticated using STR (or SNP) profiling.

### MTT assay, CCK8 assay and soft agar assay

MTT Assay: Cells were seeded in 96-well plates at a density of 500-1000 cells per well and cultured for the indicated durations. 20µL of MTT solution (5 mg/mL) were added to each well and incubated for 3 hours. The medium was then carefully discarded, and 150µL of DMSO was added to each well to dissolve the formazan crystals. Absorbance was measured at 570 nm using a plate reader.

CCK8 Assay: Cells were seeded in 96-well plates at a density of 3000 cells per well, and treated with 50nM bortezomib (Selleck, S1013) 24h later. After cultured for the indicated durations, 15µL CCK8 solution were added to each well and incubated for 3 hours. Absorbance was measured at 450 nm using a plate reader.

Soft Agar Assay: The soft agar assay was performed as previously described 30. Briefly, cells were plated in 3.5 cm dishes at a density of 500 cells per dish in soft agar and cultured for 2-3 weeks. Colonies were stained with 200µL of MTT (1 mg/mL), and the number of colonies was quantified using ImageJ software.

### Western blot

Protein extracts were prepared using RIPA buffer supplemented with 1 mM PMSF and 1x protease inhibitor cocktail (Roche Applied Science). Proteins were separated by SDS-PAGE, transferred to a membrane, and immunoblotted with the following primary antibodies: β-Actin (A3854) from Sigma-Aldrich; HADHB (ab198398), ATF6 (ab227830), XBP1 (ab220783) from Abcam; AMPKα (5831), Phospho-AMPKα (Thr172) (50081), CHOP (5554), eIF2α (5324), Phospho-eIF2α (Ser51) (3398), AKT (pan) (4691), Phospho-AKT (4060), Phospho-IkBα (2859), IkBα (4814), and GAPDH (2118) from Cell Signaling Technology. ATF4 (A21500) from ABclonal. Protein bands were visualized using a Tanon-5200 imaging system, and band densities were analyzed with ImageJ software.

### Human lung tumor samples and prognosis analysis of lung cancer patients

Clinical human lung tumor samples were obtained from Huashan Hospital, Fudan University, Shanghai, China and used for western blot analysis. Kaplan-Meier survival analysis was performed using the KM plot online tool (http://kmplot.com). We utilized the normalized microarray data from 11 lung cancer datasets (GSE3141, GSE14814, GSE19188, GSE30219, GSE31210, GSE31908-GPL570, GSE37745, GSE50081, GSE68465, GSE102287, GSE157011) downloaded from the Gene Expression Omnibus (GEO), as previously described 31. Patients were stratified by the auto-selected best cutoff for the gene of interest (*HADHB*) into high and low expression groups. Survival differences between these groups were evaluated with the log-rank test, using 10-year survival as the primary endpoint.

### Statistical analysis

Unpaired Student's t test was conducted by GraphPad Prism for statistical analysis. A p value < 0.05 was indicated statistical significance.

## Results

### *Hadhb* deficiency inhibits lung tumorigenesis in mice

**Figure 1:**
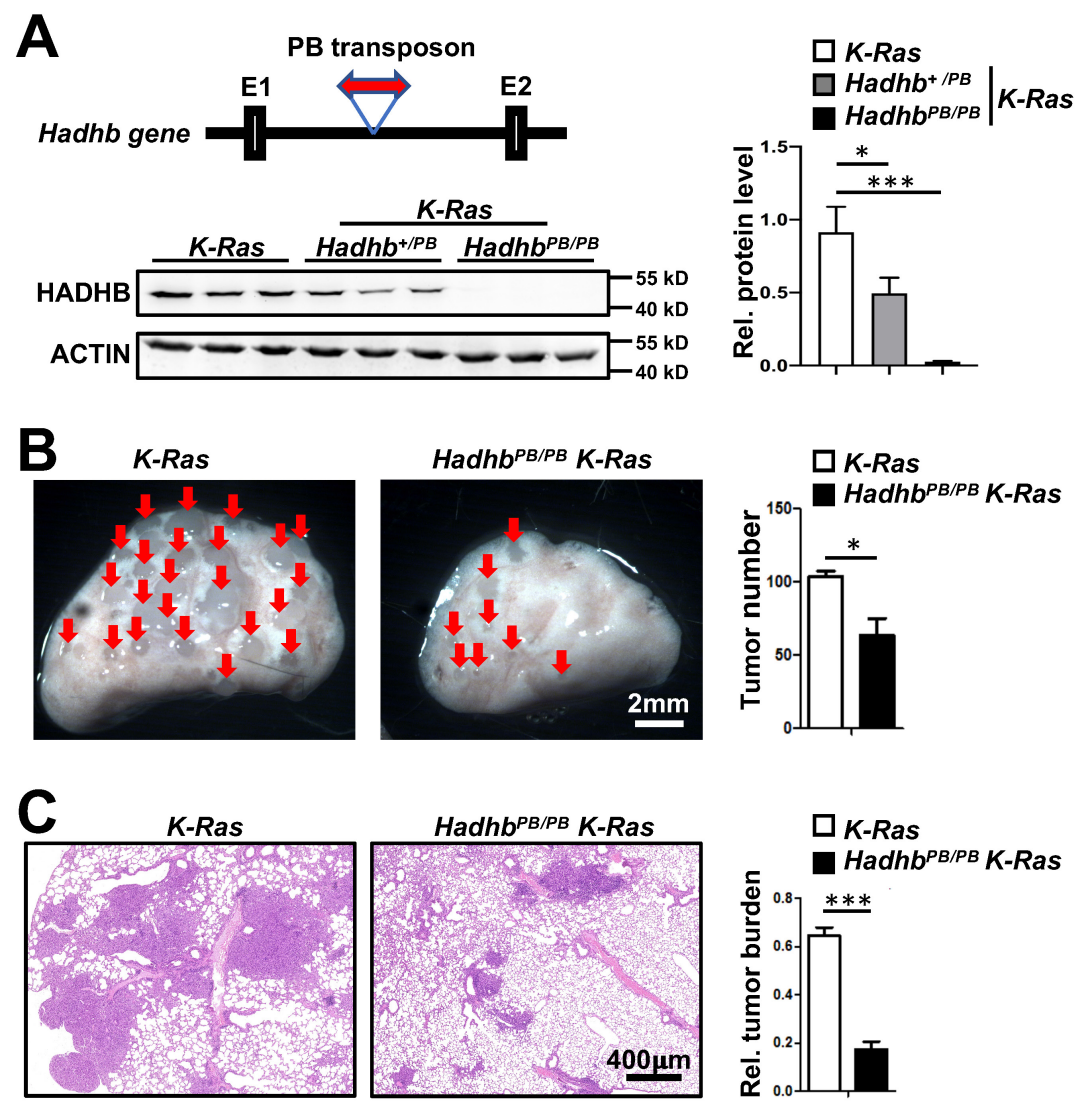
***Hadhb* deficiency inhibits lung tumorigenesis in mice.** (A) Schematic of PB transposon insertion into the *Hadhb* gene (top of left panel) and statistical analysis of HADHB protein level in lung tissue of control *K-Ras*,* Hadhb^+/PB^ K-Ras* and *Hadhb^PB/PB^ K-Ras* mice (right panel) by western blot (bottom of left panel). (B) Representative images of mouse lung tumors of *Hadhb^PB/PB^ K-Ras* and control *K-Ras* mice and statistical analyses of tumor numbers on mouse lung surface. (C) Representative histologic images of H&E-stained lung sections from mice eight weeks after tracheal instillation of Ad-*Cre* and statistical analyses of tumor burden. At least 3 mice for each genotype. Values in (A), (B) and (C) represent the means ± SD. *, *p*< 0.05 or ***, *p*< 0.001.

*Hadhb^+/PB^* mice carry a mutant *Hadhb* allele disrupted by insertion of a PB transposon in the intron between exon 1 and 2 (Fig. 1A). *Hadhb^PB/PB^* mice were viable at birth but died suddenly between 3 and 12 months of age. To investigate the role of *Hadhb* in lung cancer, we first assessed the efficacy of PB transposon-mediated disruption of *Hadhb* expression in *Hadhb^+/PB^* and *Hadhb^PB/PB^* mice. Immunoblot analysis confirmed a moderate reduction of HADHB protein in *Hadhb^+/PB^* lungs and a complete loss in *Hadhb^PB/PB^* lungs compared to wild type (WT) controls (Fig. 1A). We then induced lung tumors in *Hadhb^PB/PB^ K-Ras* and control *K-Ras* mice via intratracheal administration of adeno-*Cre* virus (see Methods). At 8 weeks post-infection, *Hadhb^PB/PB^ K-Ras* mice exhibited a significant reduction in both lung surface tumor numbers (Fig. 1B) and total tumor burden (Fig. 1C) compared to *K-Ras* controls. These results demonstrate that *Hadhb* inactivation suppresses *K-Ras*-driven lung tumorigenesis *in vivo*.

### Decreased cell proliferation and increased apoptosis in lung tumors of *Hadhb^PB/PB^ K-Ras* mice

**Figure 2:**
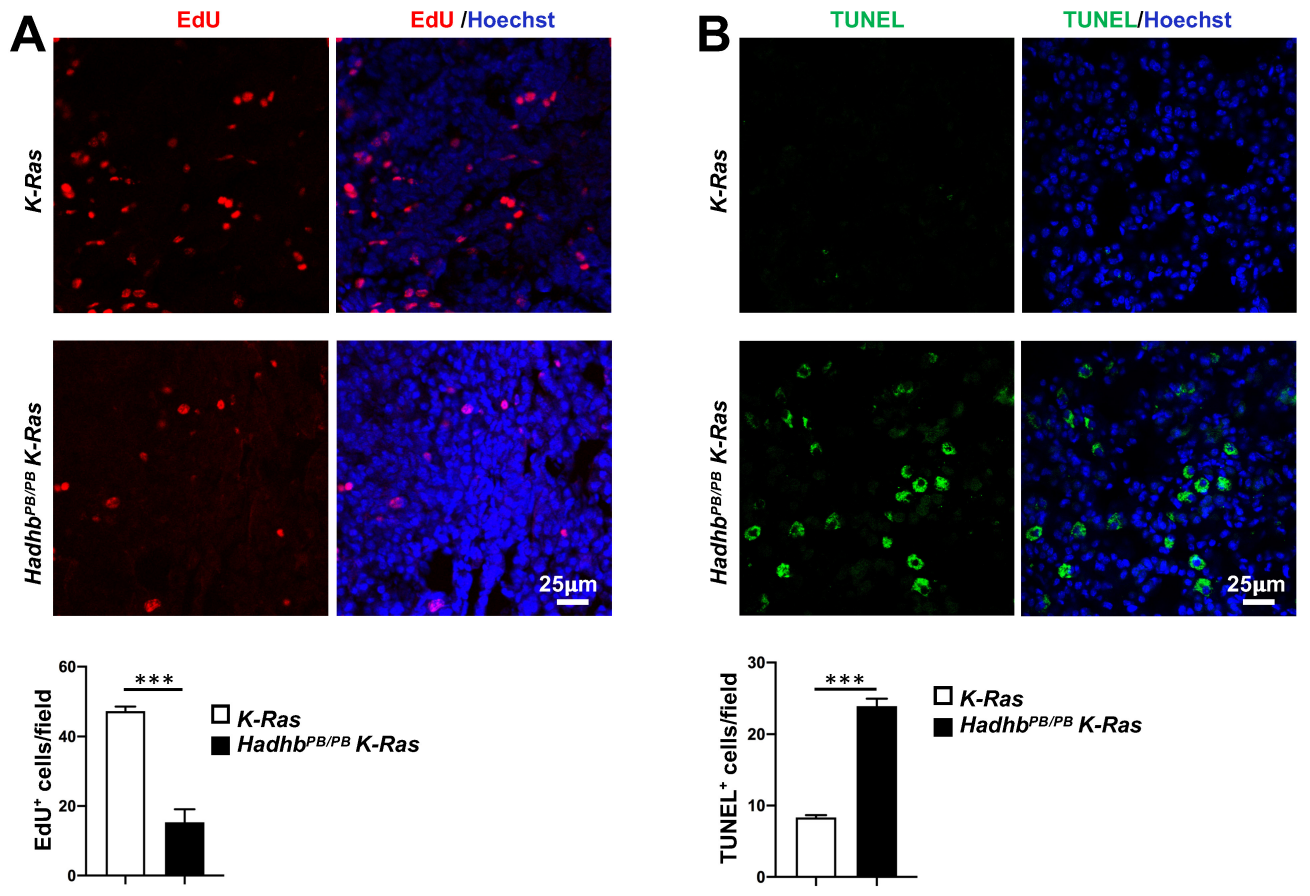
***Hadhb* deficiency suppresses proliferation and enhances apoptosis of lung tumor cells in mice.** (A) Representative fluorescent images of mouse lung sections stained for EdU-positive cells (red) in lung tumors and the statistical analyses of EdU-positive cells. (B) Representative fluorescent images of mouse lung sections stained for TUNEL-positive cells (green) in lung tumors and the statistical analyses of TUNEL-positive cells. The mouse genotypes are as indicated. At least 3 mice for each genotype. Values in (A**)** and (B**)** represent the means ± SD. ***, *p*< 0.001.

We next explored the cellular mechanisms underlying tumor suppression in *Hadhb^PB/PB^ K-Ras* mice by assessing the proliferative and apoptotic activities in lung tumors from *Hadhb^PB/PB^ K-Ras* and control* K-Ras* mice. Quantitative *in vivo* EdU incorporation assays revealed significantly fewer proliferating cells in the tumors from *Hadhb^PB/PB^ K-Ras* mice compared with those from *K-Ras* mice (Fig. 2A). Conversely, TUNEL assays showed a marked increase in apoptotic activity within the tumors of *Hadhb^PB/PB^ K-Ras* mice (Fig. 2B). These results indicate that *Hadhb* deficiency suppresses tumor growth by coordinately inhibiting cell proliferation and promoting apoptosis.

### *Hadhb* silencing impairs growth and clonogenicity of human lung cancer cells

**Figure 3:**
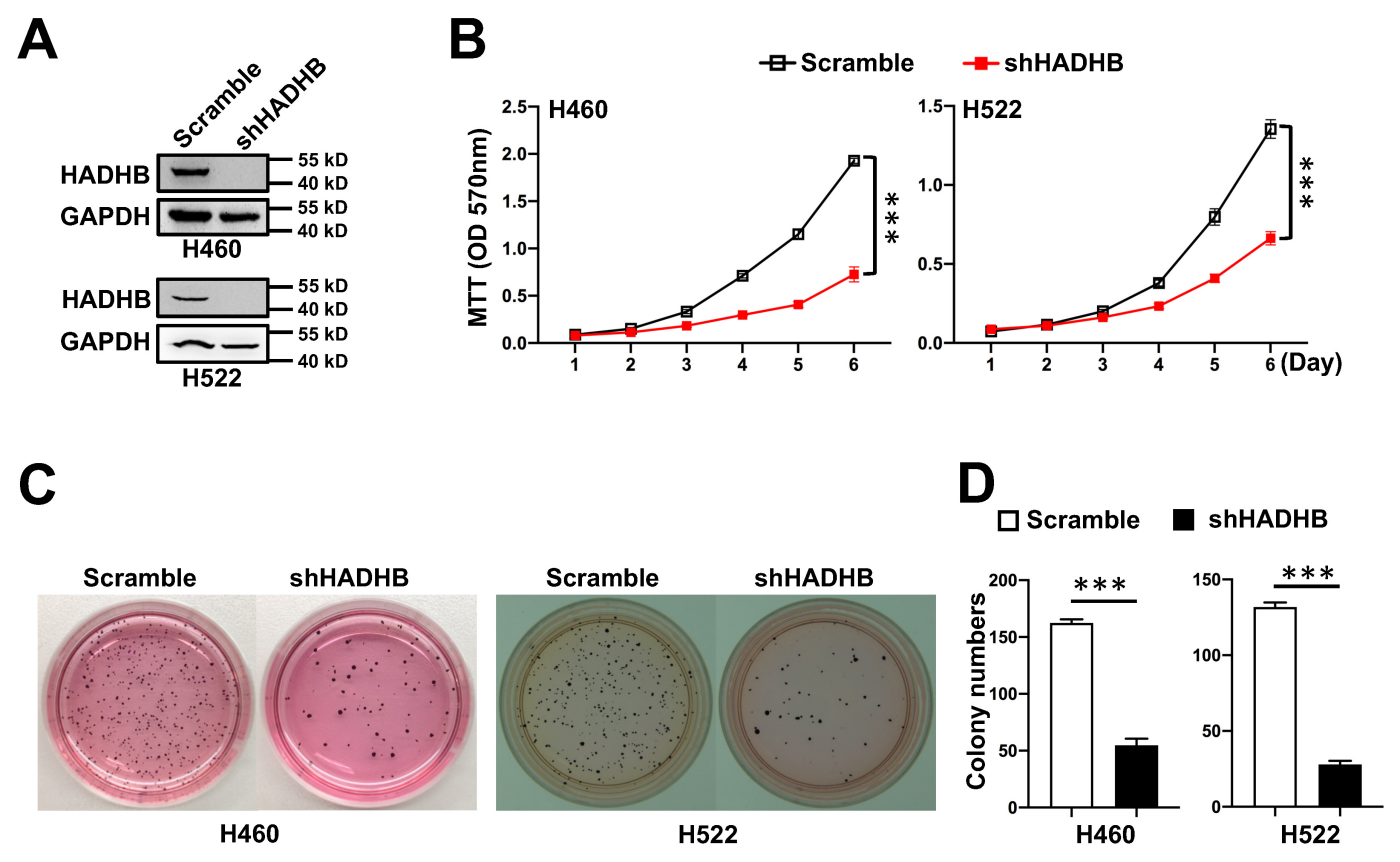
***HADHB* knockdown suppresses growth of human lung cancer cells.** (A) Representative western blot of HADHB protein levels of human lung cancer cells, expressing shHADHB and scramble shRNA. (B) The growth curves of human lung cancer cells infected with lenti-shHADHB and control viruses. Cell growth was evaluated by MTT assay. (C-D) Representative photographs of the soft agar colony formation assay for lung cancer cell lines expressing shHADHB and scramble shRNA (C), and the statistical analyses (D) of colony numbers formed in soft agar. Values in (B**)** and (D**)** represent the means ± SD. ***, *p*< 0.001.

Given the oncogenic role of *Hadhb* in murine lung tumorigenesis, we asked whether *HADHB* depletion similarly impairs human lung cancer cell growth. We silenced *HADHB* using lentiviral shRNA in two human non-small cell lung cancer cell lines (H460 and H522). *HADHB*-knockdown (shHADHB) cells exhibited significantly reduced proliferative capacity (Fig. 3A-B). Furthermore, in anchorage-independent growth assays, shHADHB cells formed dramatically fewer soft agar colonies than scramble cells (Fig. 3C-D). These findings confirm that *HADHB* depletion inhibits key malignant phenotypes in human lung cancer cells, recapitulating the *in vivo* observations.

### *HADHB* loss activates AMPK but growth suppression is AMPK-independent

**Figure 4:**
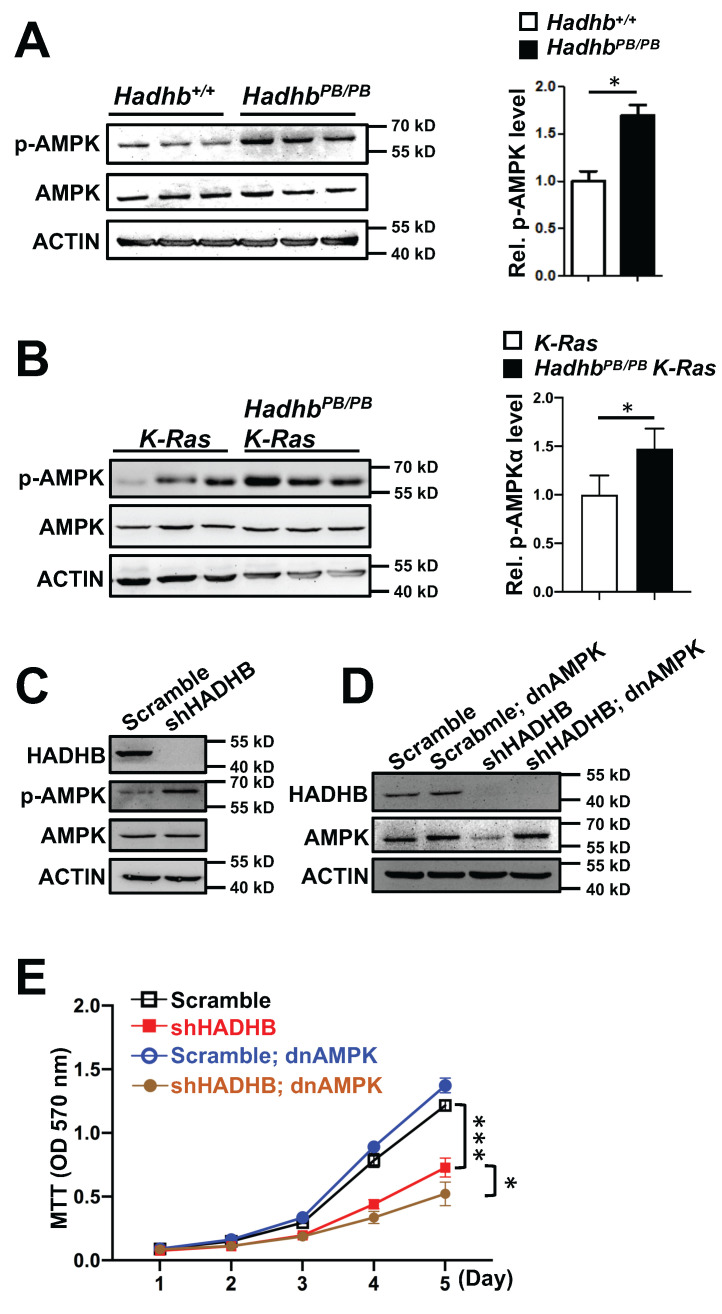
** Loss of *Hadhb* activates AMPK.** (A-B) Representative western blot and statistical analyses of AMPK phosphorylation levels in either *Hadhb^PB/PB^* mouse lung tissues (A) or *Hadhb^PB/PB^ K-Ras* mouse lung tumors (B) compared to their respective controls. At least 3 mice for each genotype. (C) Western blot analysis of the phosphorylation levels of AMPK protein in shHADHB or scramble shRNA-expressing H460 cells. (D) Western blot analysis of the HADHB and AMPK protein levels in shHADHB or scramble shRNA-expressing H460 cells infected with dnAMPK lentiviruses, respectively. (E) The growth curves of the same lung cancer cells in (D). *, *p*< 0.05 or ***, *p*< 0.001.

HADHB, the β-subunit of the mitochondrial trifunctional protein, catalyzes the final step of long-chain fatty acid β-oxidation. Given that fatty acid oxidation is a major source of cellular ATP, we hypothesized that *HADHB* depletion would disrupt cellular energy homeostasis, thereby activating the energy sensor AMP-activated protein kinase (AMPK). Consistent with this, immunoblot analyses revealed a significant increase in AMPK phosphorylation (Thr172) in both lung tissues of* Hadhb^PB/PB^* mouse and lung tumors of* Hadhb^PB/PB^ K-Ras* mouse compared to their respective controls (Fig. 4A-B). A similar increase was observed in *HADHB*-knockdown H460 human lung cancer cells (Fig. 4C), indicating a conserved metabolic stress response.

To assess if AMPK activation mediates the tumor-suppressive effects of HADHB loss, we transduced *HADHB*-depleted H460 cells with a lentivirus expressing dominant-negative AMPK (lenti-dnAMPK). Surprisingly, AMPK inhibition not only failed to rescue the proliferative defect caused by *HADHB* knockdown, but further suppressed the growth of these cells (Fig. 4D-E). This indicates that the growth suppression resulting from *HADHB* depletion occurs through an AMPK-independent mechanism.

Collectively, these data demonstrate that while *HADHB* deficiency induces energy stress and AMPK activation, the consequent tumor suppression operates independently of canonical AMPK signaling.

### *Hadhb* deficiency induces ER stress

**Figure 5:**
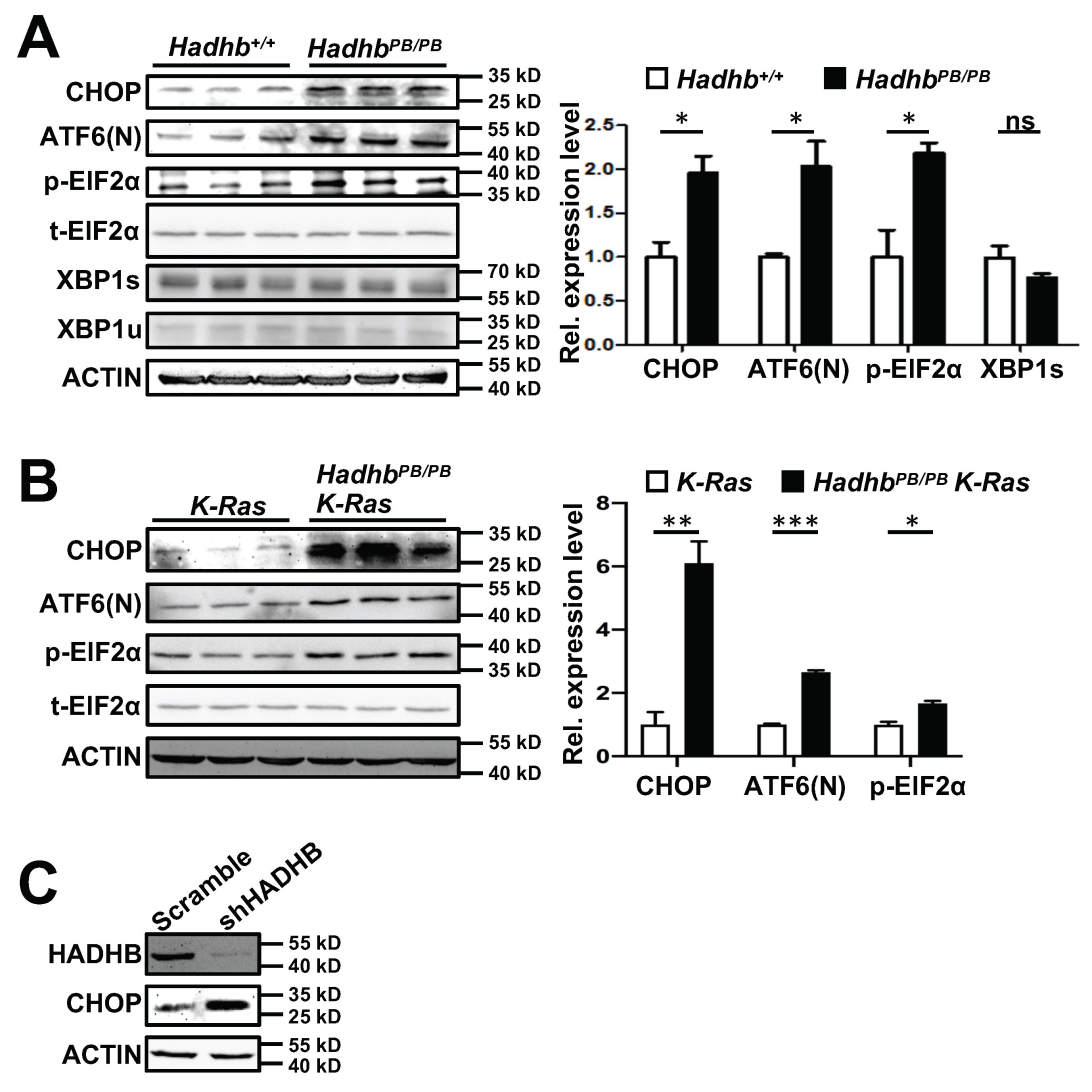
***Hadhb* deficiency induces ER stress in mouse lung tumors and normal lung tissues.** (A-B) Western blot and statistical analyses of the ER stress protein levels in either normal lung tissues from control and *Hadhb^PB/PB^* mice (A) or lung tumors from *Hadhb^PB/PB^ K-Ras* and control *K-Ras* mice (B). At least 3 mice for each genotype. (C) Western blot analysis of CHOP protein levels in shHADHB or scramble shRNA-expressing H460 cells. *, *p*< 0.05; **, *p*< 0.01; ***, *p*< 0.001 or ns, not significant.

Endoplasmic reticulum (ER) stress, often triggered by metabolic perturbations, can activate pro-apoptotic pathways. The transcription factor CHOP (C/EBP homologous protein), a key mediator of ER stress responses, drives growth arrest and apoptosis under sustained stress conditions 18. We therefore investigated whether *Hadhb* deficiency induces ER stress. Immunoblot analysis revealed significant upregulation of CHOP in both *Hadhb^PB/PB^* non-neoplastic lung tissues and *Hadhb^PB/PB^ K-Ras* tumors compared to their respective controls (Fig. 5A-B). This was also conserved in *HADHB*-knockdown H460 human lung cancer cells, accompanied by upregulation of its upstream molecule ATF4 (Fig. 5C and S1A).

To further dissect the ER stress pathways activated by *Hadhb* loss, we analyzed key signaling nodes in mouse normal lung tissues and lung tumors. Phosphorylation of eIF2α(Ser51) and cleavage of ATF6 (ATF6(N)) were markedly increased in *Hadhb^PB/PB^* mouse lung tissues and *Hadhb^PB/PB^ K-Ras* mouse lung tumors (Fig. 5A-B), whereas spliced XBP1 levels remained unchanged (Fig. 5A). These findings identify PERK-eIF2α-ATF4 and ATF6, but not IRE1-XBP1, as the dominant ER stress axes activated by *Hadhb* deficiency.

### CHOP Knock-down partially rescues the tumor suppressive effects of HADHB depletion

**Figure 6:**
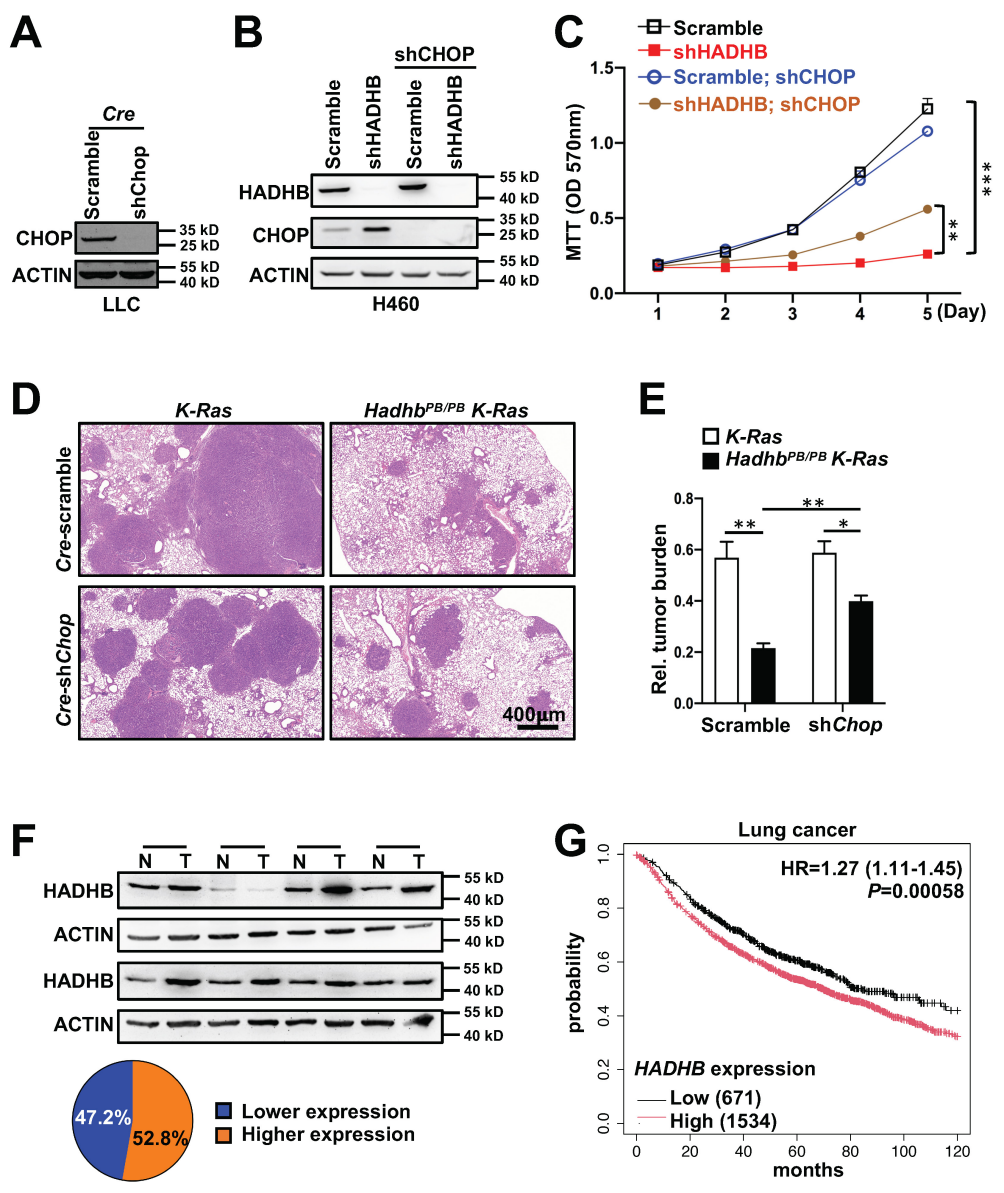
** Knock-down of *Chop* partially reverses tumor inhibition in mice and growth suppression in human lung cancer cell line triggered by *HADHB* depletion.** (A) Western blot analysis of CHOP protein levels in *Cre*-shChop or *Cre*-scramble shRNA-expressing mouse Lewis Lung Carcinoma cells. (B) Western blot analysis of the HADHB and CHOP protein levels in shHADHB or scramble shRNA-expressing H460 cells infected with shCHOP lentiviruses, respectively. (C) The growth curves of the same lung cancer cells in (B). (D-E) Illustrative histologic images of H&E-stained lung sections (D), accompanied by statistical analyses of tumor burden (E). At least 3 mice for each condition. (F) Western blot analyses of HADHB protein levels in human lung tumors and adjacent tissues, and the graph illustration of HADHB expression levels of 36 pairs of human lung cancer tissues. N: normal adjacent lung tissue, T: lung tumor tissue. (G) Kaplan-Meier survival analysis of 2205 lung cancer patients with low and high *HADHB* mRNA level in lung cancer tissues. *, *p*< 0.05; **, *p*< 0.01 or ***, *p*< 0.001.

To establish a causal role for CHOP, we performed genetic rescue experiments. We generated lentiviruses expressing Cre recombinase with either scramble shRNA (lenti-*Cre-*scramble) or *Chop*-targeting shRNA (lenti-*Cre-*shChop). The knockdown effect of lenti-*Cre*-shChop was confirmed in mouse cell line (Fig. 6A). Lenti-*Cre-*scramble and lenti-*Cre-*shChop were then delivered via intratracheal instillation to *Hadhb^PB/PB^ K-Ras* and control *K-Ras* mice, respectively. As expected, *Hadhb^PB/PB^ K-Ras* mice receiving lenti-*Cre-*scramble showed reduced tumor burden. Strikingly, this suppression was significantly reversed in *Hadhb^PB/PB^ K-Ras* mice treated with lenti-*Cre-*shChop (Fig. 6D-E). Similarly, in human H460 cells, shRNA-mediated knockdown of CHOP partially rescued the growth inhibition caused by HADHB depletion (Fig. 6B-C). These findings demonstrate that *Hadhb* deficiency suppresses lung tumorigenesis, at least in part, through CHOP upregulation.

### HADHB is upregulated in human lung cancer and correlates with poor prognosis

To evaluate the clinical relevance of *HADHB* in lung cancer pathogenesis, we analyzed HADHB protein expression in 36 paired human lung tumor and normal adjacent tissues. HADHB protein was upregulated in 52% (19/36) of tumor tissues compared to matched normal counterparts (Fig. 6F). Furthermore, Kaplan-Meier survival analysis of gene expression data from 2205 lung cancer patients revealed that low *HADHB* mRNA expression is a significant factor for good prognosis, with a hazard ratio of 1.27 (*P*= 0.00058) (Fig. 6G).

Collectively, these clinical data, combined with our functional evidence, positions *HADHB* as a pro-tumorigenic metabolic driver in human lung cancer.

## Discussion

Metabolic reprogramming is a cornerstone of cancer, with recent researches highlighting the critical role of fatty acid oxidation in supporting the bioenergetic and biosynthetic demands of rapidly proliferating tumors 32-34. While the significance of FAO enzymes in cancer is emerging, their context-dependent roles and precise mechanisms remain incompletely understood. In this study, we identify the mitochondrial trifunctional protein subunit HADHB as a novel and critical oncogenic factor in lung cancer. We demonstrate that HADHB is frequently upregulated in human lung tumors and its high expression correlates with poor patient prognosis. Using a combination of genetic mouse models and human cell lines, we establish that HADHB deficiency robustly suppresses lung tumorigenesis by inducing endoplasmic reticulum stress and activating the pro-apoptotic CHOP pathway.

Initially, we hypothesized that the tumor-suppressive effect of HADHB loss would be mediated by energy stress as fatty acid oxidation is a major source of cellular ATP. Consistent with this, we observed a conserved activation of AMPK in *Hadhb*-deficient mouse tumors and HADHB-knockdown human cancer cell lines, indicating a disruption in cellular energy homeostasis. However, the subsequent experiment using a dominant-negative AMPK construct yielded a surprising and critical insight: the growth arrest and tumor suppression occurred independently of AMPK signaling. This finding compelled us to look beyond a simple bioenergetic crisis for the primary mechanism of action. It suggests that lung cancer cells can tolerate the energy stress induced by impaired FAO, but succumb to another, more lethal consequence of HADHB deficiency.

Kao et al. reported that *Hadhb*-point mutation led to the upregulation of blood lipids in mice 6. The accumulation of toxic lipid intermediates can increase intracellular reactive oxygen species (ROS), subsequently inducing endoplasmic reticulum (ER) stress and cell death 35. Furthermore, increased palmitate rapidly increases the saturated lipid content within the ER, compromising its morphology and integrity. This disruption alters the biophysical properties of the ER membrane and impairs associated cellular functions 36. Therefore, the accumulation of toxic lipid intermediates and the impairment of ER structure and function may contribute to ER stress, potentially suppressing tumor development. Therefore, we turned our investigation to the ER. Our data robustly demonstrate that HADHB loss is a potent inducer of ER stress. We found consistent and significant upregulation of CHOP, a key mediator of ER stress-induced apoptosis, in both murine and human systems (Fig. 5), whereas AKT and NFκB signaling, which are involved in ER stress and apoptosis, remained unchanged (Fig. S1A). Furthermore, we delineated the specific UPR signaling axes involved, identifying the PERK-eIF2α-ATF4 and ATF6 branches as the primary pathways activated, while the IRE1-XBP1 arm was unaffected. This selective activation provides a more nuanced understanding of how disrupted mitochondrial FAO communicates with the ER.

The most compelling evidence for a causal role of this pathway comes from our genetic rescue experiments. The partial but significant reversal of tumor suppression upon *Chop* knockdown in the *Hadhb^PB/PB^ K-Ras* mouse model, and the concomitant rescue of cell growth *in vitro*, directly implicate CHOP upregulation as a major effector of HADHB deficiency-induced tumor suppression. The partial rescue suggests that, although CHOP is a key mediator, other AMPK- and CHOP-independent mechanisms likely contribute to the overall anti-tumor effect.

In previous reports, HADHB exhibits paradoxical roles across malignancies: it acts as a tumor suppressor in clear cell renal cell carcinoma 11, 12 and colorectal cancer 37, 38, and could be used as indicators of chemotherapy sensitivity and prognosis. However, other reports show that HADHB was frequently overexpressed in head and neck squamous cell carcinoma and high-grade lymphomas, and significantly associated with worse overall survival 13, 14. Here, we demonstrated that HADHB exerts pro-tumorigenic functions in lung cancer, and that low *HADHB* mRNA levels correlate with favorable prognosis in human lung cancers. Consistent with the tumor type-specific roles of HADHB, a previous study reported that strong CHOP activity is positively associated with good prognosis in lung cancer 39. In contrast, analysis of TCGA data revealed that high *CHOP* mRNA levels correlate with poor prognosis in kidney Renal Clear Cell Carcinoma and colon cancer patients (Fig. S2 A and B). These results suggest that the tumor-specific functions of *HADHB* may be mediated by CHOP expression levels.

Bortezomib, an ER stress inducer, is an FDA-approved drug for myeloma and has also shown activity against non-small cell lung cancer (NSCLC) and small cell lung cancer (SCLC). We found that human lung cancer cell lines with higher HADHB expression exhibited greater sensitivity to bortezomib. Following treatment, cell viability was approximately 52% in H23 cells (lower HADHB levels), whereas cells with higher HADHB levels showed reduced viability: approximately 51% in H1650, 41% in H522, 33% in H460, and 29% in A549 (Fig. S1B). These findings suggest that *HADHB* or *CHOP* expression may serve as a biomarker to stratify cancer patients for ER stress-targeted therapies.

Our integrated approach—spanning mouse genetics, human cell models, and clinical specimens—defines HADHB as a context-specific metabolic vulnerability in lung cancer. Targeting HADHB or its downstream lipotoxicity pathways could offer novel therapeutic strategies, particularly for tumors reliant on β-oxidation. Future studies should explore HADHB's interplay with lipid signaling networks and its prognostic value across cancer subtypes.

## Conclusion

Our study unveils a novel metabolic-stress axis in lung cancer. We propose that HADHB, by enabling efficient long-chain FAO, is essential for maintaining ER homeostasis in cancer cells. Depletion of HADHB disrupts this delicate balance, triggering a specific ER stress response that culminates in CHOP-mediated growth arrest and apoptosis, ultimately suppressing tumorigenesis through an AMPK-independent pathway. These findings position HADHB as a promising therapeutic target, and the HADHB-ER stress-CHOP axis as a new avenue for therapeutic intervention in lung cancer.

## Supplementary Material

Supplementary figures.

## Figures and Tables

**Table 1:** shRNA oligos.

Scramble	CAACAAGATGAAGAGCACCAA
shHADHB	CGTTAGCCAAACCCAATATAA
shCHOP(human)	TGAACGGCTCAAGCAGGAAAT
shChop(mouse)	GATTCCAGTCAGAGTTCTATG

## Data Availability

The datasets generated during and/or analyzed during the current study are available from the corresponding author on reasonable request.

## Abbreviations

ER: endoplasmic reticulum
FAO: fatty acid β-oxidation
GEO: Gene Expression Omnibus
MTP: mitochondrial trifunctional protein
UPR: unfolded protein response
