# Supplementary material for: Hadhb Deficiency Inhibits Lung Tumorigenesis Via Activating ER Stress
Source: J Cancer. 2026 May 1;17(5):990–1001. doi: 10.7150/jca.130384 (PMC13189847; doi:10.7150/jca.130384)
Supplement: Supplementary file 1 — Supplementary figures. [file jcav17p0990s1.pdf]

Supplemental information

Figure S1

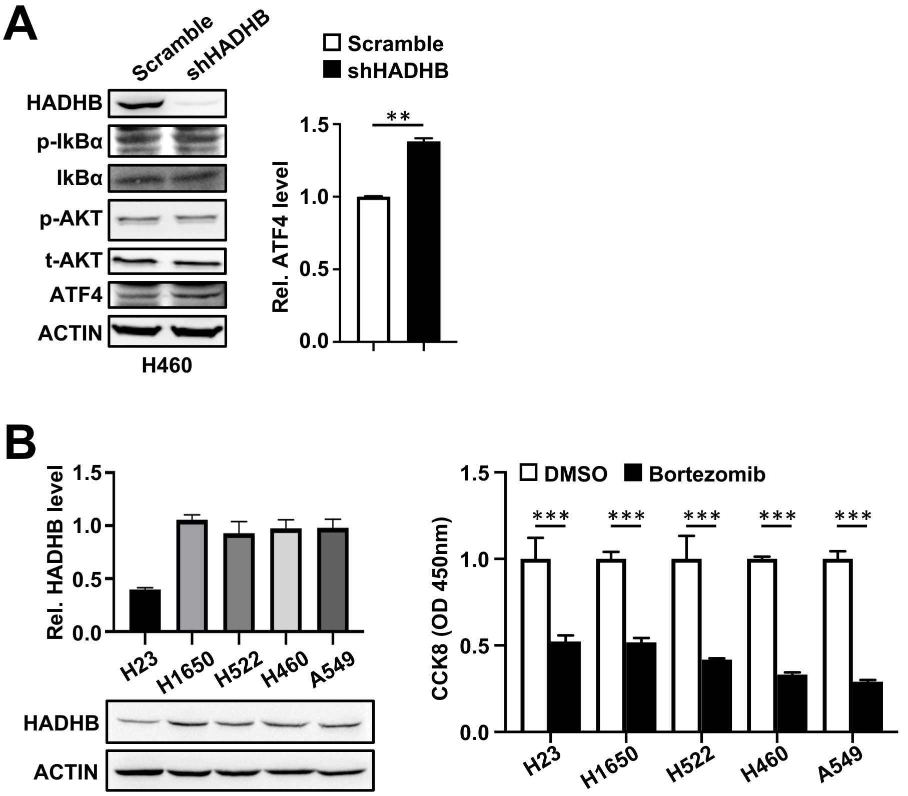

Fig. S1 The effects of HADHB protein levels on downstream targeting molecules of the PERK and ATF6 pathways and sensitivity to bortezomib treatment in human lung cancer cell lines. (A) Western blot analysis of HADHB, p-IkBα, IkBα, p-AKT, AKT, and ATF4 proteins (left) and statistical analyses of ATF4 protein (right) in shHADHB and control H460 cells. (B) Western blot analysis of HADHB expression (left) and assessment of bortezomib sensitivity (right) in five human lung cancer cell lines. ACTIN serves as a loading control. **, *p*< 0.01; ***, *p*< 0.001.

Figure S2

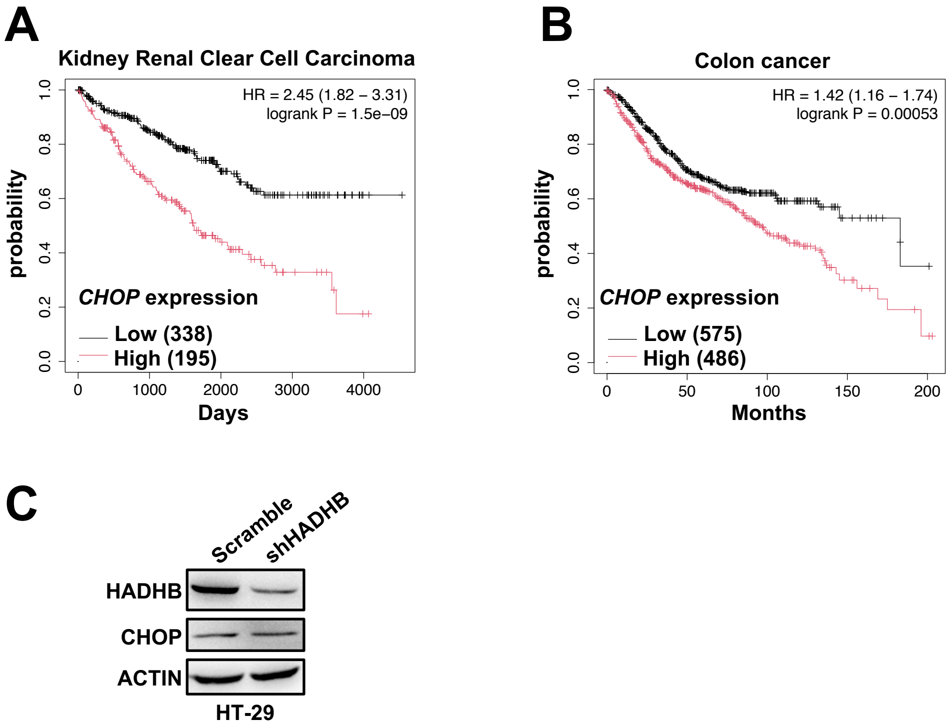

Fig. S2 High *CHOP* mRNA levels correlate with poor prognosis in kidney Renal Clear Cell Carcinoma and colon cancer patients. (A-B) Kaplan-Meier overall survival analyses of 533 kidney Renal Clear Cell Carcinoma patients (A) and 1061 colon cancer patients (B) with low and high *CHOP* mRNA level. (C) Western blot analyses of HADHB and CHOP proteins in shHADHB and control HT-29 cells. ACTIN serves as a loading control.
